# A developed model of cancer patients participation in intravenous chemotherapy safety

**DOI:** 10.18632/oncotarget.20986

**Published:** 2017-09-18

**Authors:** Zeng Na, Yan Qiaoyuan, Wang Binghan, Zhu Qin, Chen Yue, Peng Xin, Tan Cuilian, Yao Cheng

**Affiliations:** ^1^ College of Medical Science, China Three Gorges University, Yichang 443000, Hubei, China; ^2^ Union Hospital, Tongji Medical College, Huazhong University of Science and Technology, Wuhan 430022, Hubei, China; ^3^ School of Nursing, Tongji Medical College, Huazhong University of Science and Technology, Wuhan 430030, Hubei, China; ^4^ First Affiliated Hospital, Chongqing Medical University, Yuzhong 400000, Chongqing, China; ^5^ Public Health Department, Tongji Medical College, Huazhong University of Science and Technology, Wuhan 430022, Hubei, China

**Keywords:** patient participation, safety, model, intravenous chemotherapy, cancer

## Abstract

How to reduce intravenous chemotherapy-related adverse reactions of cancer patients is one focus of clinical work. Nowadays, patient for patient safety (PFPS) is an important component of hospital safety management and can contribute to a reduction in the rate of adverse events following intravenous chemotherapy of cancer patients. To guide and evaluate cancer patients participate in intravenous chemotherapy, we explored a scientific and practical model of cancer patients participation in intravenous chemotherapy safety. which can also guide nurse practitioners (NPs) practice. Based on a literature review and analysis of chemotherapy-associated adverse events from two large comprehensive hospitals, combined with the existing strategies for PFPS, the model of cancer patients participation in intravenous chemotherapy safety was drafted. Then we conducted two rounds of the Delphi-method questionnaire to revise the model. The two rounds Delphi questionnaire survey had a response rate of 82.36%. The authoritative coefficient was 0.87 and the coordination coefficients were 0.165 and 0.214, respectively. The proposed safety model included 3 first-order indicators, 8 second-order indicators, and 41 third-order indicators, including content of patients participation, responsibilities of medical personnel to assist cancer patients participation, and suggestions for guaranteeing implementation. Many NPs practice in a medical setting where cancer patients for patient safety behavior are blurred. The model of cancer patients participation in intravenous chemotherapy safety can guide NPs in their practice of promoting PFPS among cancer patients intravenous chemotherapy.

## INTRODUCTION

Medication errors, defined as preventable events that may lead to inappropriate medication use or patient harm, are a serious and common threat to cancer patients [1]. It was reported that there were 421 million hospitalisations in the world annually, and approximately 42.7 million adverse events [2] and medication errors cost an estimated 42 billion USD annually [3]. Unsafe medication practices and medication errors are leading causes of injury and health care associated harm around the world [4, 5]. Intravenous (IV) chemotherapy, which can provide rapid drug delivery to the body of cancer patients, thus initiating a rapid systemic response, is essential treatment for most cancer patients [6]. However, IV chemotherapy is a complex process requiring proper drug preparation before administration to the patients and errors occurring at any stage can cause harmful clinical outcomes to the patients, which may lead to morbidity and mortality [7, 8]. IV chemotherapy presents special dangers because: (1) many drugs have a narrow therapeutic index; (2) are toxic even at therapeutic dosages; (3) chemotherapy regimens are highly complex; and (4) cancer patients are a vulnerable population with little tolerance [9]. Accordingly, research into the IV chemotherapy safety of cancer patients is required. How to ensure IV chemotherapy safety, avoid increased hospitalization costs, and improve patient quality of life are therefore the focus of our study.

As a participant in the entire process of diagnosis, examination, treatment, and care, patients can provide detailed and accurate information to medical staff because they represent the main body of health maintenance [10, 11]. The World Patient Safety Alliance points out that patients should become active participants in medical safety rather than passive recipients [12]. Researches showed that frequently observe, report, and intercept errors and patients who are more involved with their care tend to get better results [13, 14]. Chemotherapy patients may be particularly qualified to get involved in error prevention as they often experience recurrent procedures and intense episodes of care and thus develop expertise regarding treatment administration [15]. Studies indicate that cancer patients increasingly want to be involved in their care and want to know more about the medications that have been prescribed for them [16]. And several researches have explored strategies for cancer patients to participate in chemotherapy safety, for example, Agency for Healthcare Research and Quality (AHRQ) putted forward “20 Tips to Help Prevent Medical Errors: Patient Fact Sheet” to help patients participate in self-safety management in the course of medication [17]. The Ministry of Health (MOH) has adapted the concept of patients and community engagement and empowerment into its 9th strategic plan (2015-2020) [18]. 2016 Updated American Society of Clinical Oncology/Oncology Nursing Society Chemotherapy Administration Safety Standards put forward that patients are required to know about planned duration of treatment, schedule of treatment administration, drug names and supportive medications, drug-drug and drug-food interactions, and plan for missed doses to promote patient participation in self-safety management [19].

However, although researches in this area have proposed workable solutions for cancer patients with respect to drug safety, the nature of participation remains narrow and lacking in systematicness and there are no strategy targeted for the safety of IV chemotherapy of cancer patients. Research on health engagement has proved challenging, however, because of established power differentials between patients, care providers, and researchers [20]. Chemotherapy patients are more challenging in participating in self-safety management because of physical weakness and the particularity of treatment [21–23]. In this study, based on a literature review and the factors contributing to IV chemotherapy-related adverse events: medication errors, peripheral venous leakage, checking lax, and poor physical condition of the cancer patient, combined with some existing patient engagement safety strategies and ultimately using the Delphi-method questionnaire, a classical study was designed that used an iterative questionnaire to measure consensus among individual responses without interaction. We then developed a model for the cancer patient participation in IV chemotherapy safety to address the shortcomings in cancer patient involvement in IV chemotherapy safety management and to promote the spread and implementation of the subject as a further step.

## RESULTS

For research purposes, we divided the 68 relevant articles identified in the literature search into four categories (Table 1). The 40 IV chemotherapy-related adverse event factors were also categorized (Table 2).

**Table 1:** Literature categories

Category	Number
Patient cognition	23
Medicine safety	25
Involvement strategy	9
Medical staff-related	11
**Total**	68

**Table 2:** Categorization of IV chemotherapy adverse event factors

Category	N	Constituent ratio (%)
Medication error	11	27.5
Iatrogenic vascular skin injury	10	25.0
Venous indwelling catheter-related incidents	7	17.5
Accidents	6	15.0
Medical material incidents	4	10.0
Transcription errors	2	5.0
Total	40	100.0

### Degree of enthusiasm and authority of the expert panel

In this study, 17 questionnaires were distributed in two rounds, and 14 were returned in each of round. The effective return rate was therefore 82.36%, and a total of 20 amendments were proposed. The experts were shown to have a high degree of enthusiasm for the study subject. The degree of expert authority was determined by two factors, the basis of expert judgment, represented by Ca; and the familiarity of experts with relevant issue, represented by Cs. The extent of the authority of experts was mainly determined by self-evaluation and by using the arithmetic average value of the expert judgment coefficient and degree of familiarity coefficient, that is Cr = (Ca + Cs) / 2. In this study, the extent of the authority of experts was Cr = 0.87 (Cr ≥ 0.70 represents acceptable reliability).

### Degree of centralization and coordination of expert advice

In two rounds of expert consultation, two experts raised an objection to the first-order indicators. The 4 third-order indicators with coefficient of variations > 0.25 in the first round were discussed and modified according to the experts’ opinion. There was only one high coefficient in the second round, with a value of 0.43, indicating that the experts’ opinions on this items differed greatly. Therefore, the item “encourage patients to participate in ECG reading content” was omitted following discussion. The cooperation coefficient (Kendall's W) was used to analyze the data consistency of the third-order indicators of experts’ opinions after two rounds of consultation (Table 3).

**Table 3:** Cooperation coefficient (Kendall's W) of third-order indicators of experts’ opinions

Time	Indicator	W	X^2^	P
1	Third-order indicators	0.165	79.25	<.001
2	Third-order indicators	0.214	95.16	<.001

### Confirmation of patient participation in IV chemotherapy safety model accepted suggestions of first and second round delphi-method questionaire

adjusted first-order indicator “Quality Assurance” into “suggestions for guaranteeing implementation”, deleted “forms of patient participation” tuning second- and third-order indicators statements as well;

changed some contents of 12 third-order indicators to make them closer to the clinical practice. Such as during the process of chemotherapy infusion, if the patient feel any unwell “shut down the infusion” revised into “immediately alerts a nurse”;

added 2 items of third-order indicators, when “cooperate with nurses to evaluate the risk of falling and of pressure sores” added, responsibilities of medical staff added likewise;

deleted 3 second-order indicators and 2 third-order indicators, considering they are too hard for patients to complete or they may disturb nomal nursing;

There are still some suggestion we objected with team discussion.

After two rounds of Delphi-method questionnaire combined with discussions in the expert coordination team, the theoretical model of the cancer patient participation in IV chemotherapy safety was confirmed with 3 first-order indicators: content of patient participation, responsibilities of medical personnel to assist patient participation, and suggestions for guaranteeing implementation; 8 second-order indicators and 41 third-order indicators. The first-order and second-order indicators of the model are presented in Table 4, while the third-order indicators are presented in Table 5.

**Table 4:** Model of cancer patient participation in IV chemotherapy safety (first- and second-order indicators)

First-order indicators	Second-order indicators
1 Content of patients participation	1.1 Participation in decision making
	1.2 Caring presence
	1.3 Demands participation
2 Responsibilities of medical staff	2.1 Participation in decision making
	2.2 Caring presence
	2.3 Requesting help
3 Suggestions for guaranteeing implementation	3.1 Suggestions for management
	3.2 Suggestions for safety culture

**Table 5:** Model of cancer patient participation in IV chemotherapy safety (third-order indicators)

Third-order indicators
1.1.1 Patients must accurately report physical and psychological discomfort
1.1.2 Patients must actively report adverse effects in previous treatment and nursing (such as drug use, blood transfusion, and surgery)
1.1.3 Patient and attending physician make a joint decision about chemotherapy
1.1.4 When the physician changes the type and dose of chemotherapy, the patient understands the reason and receives basic information about the new chemotherapy drug(s)
1.1.5 Patients understand the catheterization methods (includes outer periphery trocar, outer periphery of central venous catheter, clavicle central venous, and implantable venous port) surgeries, indications, advantages, disadvantages, and costs
1.1.6 Before discharge, the patient informs the doctor of relevant circumstances at home
1.2.1 Patients ask nurses for chemotherapy cards to learn the names of chemotherapy drugs, infusion requirements, and common adverse reactions
1.2.2 Under the guidance of the nurse, the patient masters conventional nursing methods related to the venous catheter used and implements routine maintenance
1.2.3 The patient, together with the nurse, checks the name, hospital number, and drug name card information and infusion order of chemotherapy drugs before transfusion and when replacing medicine bags or bottles
1.2.4 During the process of chemotherapy infusion, the patient reports and abnormality in puncture site of intravenous infusion such as redness, swelling, or pain, and immediately alerts a nurse
1.2.5 During the process of chemotherapy infusion, the patient observes whether infusion is smooth; if the drip rate appears to slow or stop, the nurse will be called immediately
1.2.6 If there is leakage of chemotherapy drugs, patients should actively cooperate with the nurse or take such medication as the doctor orders. At the same time, the patient should raise his or her affected limb to reduce edema and avoid straining the affected limb to avoid pressure
1.2.7 Patients are concerned about their test results, and ask the doctor or nurse to explain them if in doubt
1.2.8 Patients should cooperate with nurses to evaluate the risk of falling and of pressure sores; high-risk patients must ensure to implement the nursing instructions
1.2.9 Patients adhere to a light, nutritious diet, consume more than 1500 ml of water daily, and ensure reasonable arrangements for activity and rest
1.2.10 If the physical condition of the patient does not allow self-care, a reliable friend or relative can be chosen as a facilitator
1.3.1 If the patient experiences any discomfort during chemotherapy, he or she can report the situation to the attending physician and the nurse at any time
1.3.2 When the patient's mood is low, he or she can seek help from the doctors and nurses
1.3.3 During treatment and care, if the patient identifies any errors, he or she can communicate directly with the involved parties; if necessary, the head nurse should be informed
2.1.1 Medical staff actively establish a relationship of mutual trust with patients and encourage patients to participate in self-management and safety
2.1.2 Medical staff guide patients to disclose past medical history voluntarily, and listen to it with patience
2.1.3 According to the education and age characteristics of the patient, an appropriate choice of communication should be used for patients and their families to explain the advantages, disadvantages, and costs of alternative treatments and to work together with patients to determine the final plan for chemotherapy
2.1.4 Before changing chemotherapy, the attending physician should explain the reasons to the patient and seek his or her consent
2.1.5 Primary nurses assess the patient's risk of exosmosis and explains the advantages and disadvantages, indications, costs, and other issues of alternative catheter methods to help patients choose their own catheter type
2.1.6 Primary nurses and doctors work with the patient to develop a discharge program based on the patient's condition and understanding of the actual situation in the patient's home.
2.2.1 Primary nurses issue chemotherapy drug information cards to patients and explain to and remind the patients of the do's and don’ts, according to the patient's chemotherapy regime
2.2.2 Primary nurses explain the catheter to the patient and teach the patient to observe whether it is normal or not, according to catheter method
2.2.3 Before chemotherapy infusion, primary nurses explain the names and doses to patients and their families. Before the infusion or replacement of the drug bag (bottle), the nurse invites the patients to check patient's name and hospital number
2.2.4 Primary nurses explain phlebitis, venous thrombosis, and infusion leakage to patients and their families and encourage patients and family to learn self-observation.
2.2.5 During the process of chemotherapy infusion, the primary nurses increase inspections, raise vigilance, and deal with issues such as transfusion obstacle in a timely way.
2.2.6 Primary nurses assess the risk of falls and pressure sores. For the patient at high risk, the nurses perform special identification at the bedside, provide nurse's orders cards, and teach each patient a self-care routine
2.2.7 Primary nurses should issue and clarify health education prescriptions to patients based on the disease
2.3.1 When a patient is in doubt, medical staff should patiently listen to him or her and give reasonable explanations
2.3.2 Medical personnel should try to meet reasonable needs of patients
2.3.3 Encourage patients to stand up for their rights and report error events
3.1.1 We recommend that chemotherapy departments establish a quality control group for the safety of patients receiving intravenous chemotherapy, with a nurse team leader as the quality control head. Chemotherapy patient self-efficacy and other related questionnaires can be used to evaluate the effect of this strategy
3.1.2 We recommend that chemotherapy departments arrange chemotherapy drug educational training to enhance the professional knowledge and ability of nurses
3.1.3 We recommend that chemotherapy departments arrange psychological counseling training to enhance medical staff ability to assist patients in addressing their psychological problems
3.2.1 We recommend that chemotherapy departments promote the meaning and importance of patients in patient safety, and encourage medical staff to guide patients involved in safety management
3.2.2 We recommend that chemotherapy departments improve health education to meet the needs of patients for disease-related knowledge
3.2.3 We recommend that hospitals establish an incentives policy for patients in patient safety, and that patients are not dominated by medical personnel but instead cooperate with medical staff

## DISCUSSION

### Significance of the cancer patient participation in IV chemotherapy safety model

Cancer patients are at the center of medical services. To ensure *cancer* patient safety should be the permanent objective of hospital management. The administration of medication represents one of the main methods of medical treatment, although its universal usage and varied classification contribute to its high risk of adverse events. Given the specificity of IV chemotherapy medication, not only can its objective and usage be incorrect, but adverse reactions may occur within the therapeutic dose including digestive disturbances, myelosuppression, and alopecia, as well as errors arising from physical or psychological intolerance. IV chemotherapy (systemic therapy) is the main route of administering chemotherapy. As a consequence of the significant infusion quantity required, IV chemotherapy can require extended administration periods. With fewer nurses available during certain periods (e.g., at night), coupled with various demands such as infusion order, drip speed, and light sensitivity of the medication, administration of IV chemotherapy is associated with higher risk than normal infusion. Furthermore, given the likelihood of cancer patients to be in worsened condition, the safety of IV chemotherapy has been an important consideration for many years. Our research is based on theory combined with literature review and undesirable chemotherapy events analysis, which has been subjected to professional consultation. The cancer patient participation in IV chemotherapy safety model is composed of patient participation, the duties of medical staff, and the recommendations for guaranteeing implementation. Cancer patients’ participation is given in sequence of time and importance, covering the entire process of pre-chemotherapy, chemotherapy, and intervals between chemotherapy. Considering that the current theory of patient involvement is insufficient in hospitals and that deficits in patient awareness and provision of health information exist, we highlighted the duty of medical staff with the hope of their effective cooperation with patients in the model. Apart from this, for the current hospital status and management system, suggestions to further guarantee the safety of cancer patients’ participating in IV chemotherapy are proposed. This model can give a integrated guidance for patients and medical staff for specific implementation of participating in IV chemotherapy safety, and can be adjusted with hospital or department reality. It is expected to make much safer for cancer patients.

### Reliability of results

The research is based on a substantial literature review with analysis of 40 hospital chemotherapy adverse events at two tertiary hospitals of China, and a summary of the classification of safety events during IV chemotherapy involving patient participation, combining King's goal-attaining theory with the theory of patient safety of interactive involvement. Following discussion by the expert coordination group, a model for safety of cancer patients participating in IV chemotherapy was formulated. The experts performed well in two rounds, with a questionnaire response rate of 82.36% and an authoritative coefficient of 0.87, indicating the high authority of the experts in this field. After two rounds of expert consultation, the coordination coefficient increased from 0.165 to 0.214, indicating that the divergence of experts’ opinion decreased and that they tended to be in agreement. The evaluation content of the third-order indicators included not only appropriateness but also the grade of feasibility. The items with an average score less than 3.5 and variable coefficient greater than 0.25 required modifying. The draft of the model, which has a solid theoretical foundation, was modified for the clinical setting by the final expert consultation.

### Scientificity of indicators

The first-order indicators of cancer patient involvement content are the cornerstone of the entire model. The second-order indicators include decisive involvement, caring involvement, and complaint involvement. Ye [24] have previously provided an explanation of these three definitions. Throughout the process of IV chemotherapy, from diagnosis to rehabilitation nursing, decision making, caring, and complaints, cancer patients play an important role and their effective participation can reduce the risk of adverse events and further promote patient safety. The third-order indicators show how cancer patients can get involved and at which stage to participate. This content is clear and has good operability. However, due to the expertise of the medical field, it is not equivalent to the health knowledge of cancer patients and medical staff. Some research has shown that patients are unwilling to ask questions that they think might challenge the ability of medical staff to avoid causing annoyance and damaging the relationship and trust between medical staff and patients, reportedly the key impediment to patient participation [25–27]. Furthermore, because medical staff overload and the differences between patients in terms of literacy, it can be difficult for doctors and nurses to communicate satisfactorily with patients, thus preventing patient participation in safety management. In view of these factors, the model also recommended that medical staffs should play an important role in assisting cancer patients participate in decision making, self caring, supervision during the process of IV chemotherapy. In addition, according to the current hospital situation of patient participation in patient safety, an important guarantee of safety management is to propose an organizational system and safety culture to ensure the smooth progress of activities that cancer patients participating in IV chemotherapy safety.

### Limitations of study

There were some limitations to our study. First, since the date of IV chemotherapy adverse events analysis were only derived from two hospitals, it may be considered insufficient. Second, the panel of experts did not include healthcare professionals other than nurses, who might have different views on the subject. We intentionally invited only nurses because the most contact with cancer patients during the progress of IV chemotherapy are nurses, and this could be considered a limitation of the Delphi survey. Lastly, this study describes only one theoretical model which has not been implemented in a hospital setting, meaning that further research is required.

## MATERIALS AND METHODS

### Formation of the draft model

The reference standards of a safety model for cancer patients participating in IV chemotherapy were developed using relevant literature identified using Google scholar, Wanfang Data, CNKI, PubMed, Elsevier, Ovid and other databases to retrieve PFPS and patient participation data (*keywords: “cancer”, “cancer patient”, “chemotherapy”, “patient participation”, “Patients for patient safety”, “patient participation”, “patient involvement”, “Patient engagement”, and “patient-centered care”*). We also searched the relevant literature on drug safety in chemotherapy (*medication safety, patient safety, and drug therapy*) to review the progress and current status of cancer patients participation in IV chemotherapy safety management and to further identify the research issues. From June 2016 to December 2016, we identified 91 relevant articles and selected 68 that focused on cancer patients participation in their own medical safety. We referred to the relevant literature, taking full account of the patients and their families willing to participate [28, 31], and considered barriers and facilitators to chemotherapy patients’ engagement in medical error prevention [29, 30]. We also referred to other researchers’ study of patient involvement strategies to efficiently avoid chemotherapy errors [31–33], as well as to instructions on ensuring safe medical treatment by the National Patient Safety Foundation [34]. The literature was reviewed to identify relevant material on cancer patients participation before, during, and after IV chemotherapy. Next, we performed an analysis of the causes of undesirable chemotherapy events by collecting undesirable events from the hospital records of two tertiary hospitals, Tongji Hospital and Union Hospital (affiliated to Tongji Medical College, Huazhong University of Science and Technology) between January 2009 and December 2016, and used attribution theory and general systems theory to analyze the causes of these events. The literature was reviewed to identify relevant material on cancer patients participation before, during, and after IV chemotherapy.

Theoretical guidance on developing a safety model for cancer patients participation in IV chemotherapy was obtained using King's standard theory and an interactive theoretical model of participant in patient safety [35]. The principles for construction of the model for cancer patients participation in IV chemotherapy safety included: *Feasibility principle*: execution and content of patient participation in IV chemotherapy safety is accessible to patients, and cooperation by medical personnel is available; *Protective principle*: patients have the right to know and select their treatment or nursing plan, and to maintain their own privacy. Medical personnel are duty bound to protect patients’ right when participating in IV chemotherapy safety; *Interactive principle*: patients participation in safety is a process during which medical personnel and patients interact. Therefore, coordination and cooperation between nurses and patients are required; *Safety-first principle*: patients should exercise their rights legitimately and fulfill their obligations conscientiously, without over-emphasizing their rights (which can affect normal treatment and nursing), to avoid excessively affecting medical safety.

Through the literature reading and undesirable chemotherapy events analysis, under the guidance of the four basic principles, we found out where and how patients participate around the chemotherapy process, then developed the model draft of cancer patients participation in intravenous chemotherapy safety.

### Model revision

Using the Delphi-method questionnaire, according to the personal understanding and awareness of the experts involved, the contents of each part of the proposed cancer patients participation in IV chemotherapy safety model included an evaluation of whether the indicators and descriptions were appropriate. The feasibility of the third-order indicators was also assessed and amendment was proposed. Finally, the consultative results were analyzed and the model was revised.

### Expert coordination team

The expert coordination team was composed of four members, including two deputy director nurses working in clinical nursing for many years, a charge nurse with a high standard of scientific accomplishment, and a postgraduate nursing student. The team members were primarily responsible for content discussions and model decisions; preparing a consultative table of experts; and analyzing, discussing, and revising experts’ suggestions and results.

### Inclusion criteria and composition of consultant panel

Participants were selected by field of expertise, willingness to participate, participation time requirements, and other conditions. A total of 14 clinical nursing experts with intermediate and higher professional titles were selected, all working in nursing safety management, oncology nursing management, or clinical oncology nursing for more than 10 years at one of three hospitals in Wuhan, China. Furthermore, three education experts with more than 10 years of nursing education were selected from Tongji Medical College, Huazhong University of Science and Technology, and Wuhan University School of Medicine, resulting in a total of 17 panel members. All consultants were female, aged 45.00 ± 6.79 years and with work experience of (26.50 ± 7.45) years.

### Delphi survey process

Questionnaires were distributed and retrieved by email and by on-site survey. The contents of the first round of consulting questionnaires included the questionnaire description (introduction to the research background, research objectives, and participant statement and requirements). A questionnaire on participants detailed was also included to collect information on age, educational background, position, title, main working areas and working years, judgment basis of the consultants, and the degree of familiarity with the research topic. The judgment basis included an assessment of the degree of knowledge about participation in patient safety, IV chemotherapy, the current status of the hospital service, and the safety culture of the hospital. These four dimensions were graded as high, medium, and low degree. The content evaluation of cancer patients participation in IV chemotherapy safety included 4 first-order indicators, 11 second-order indicators, and 41 third-order indicators. For the 41 third-order indicators, the consultants were required to evaluate whether the contents of indicators were appropriate at all levels and to propose possible amendments. Furthermore, a feasibility evaluation was required for the third-order indicators on a scale of 1–5, where 1 point corresponds to least practicability. According to consultant opinions and statistical results, the first round questionnaire was amended and supplemented to produce the second round questionnaire, which was distributed by post. After the two survey rounds, all indicators that matched the conditions of mean value of feasibility >3.5 and coefficient of variation <0.25 were adopted, combined with the experts’ text opinions, followed by review and discussion by the expert coordination team.

### Statistical analysis

Excel 2007 and SPSS 20.0 were used for data processing and analysis. The descriptive analysis of content included frequency, mean, standard deviation, coefficient of variation, authority of expert consultation, and coordination coefficient of the outcome, using Kendall's W based on the calculation formula.
